# The CASCADE trial: effectiveness of ceramic versus PEEK cages for anterior cervical discectomy with interbody fusion; protocol of a blinded randomized controlled trial

**DOI:** 10.1186/1471-2474-14-244

**Published:** 2013-08-16

**Authors:** Mark P Arts, Jasper FC Wolfs, Terry P Corbin

**Affiliations:** 1 Department of Neurosurgery, Medical Center Haaglanden, PO Box 432, 2501, CK The Hague, The Netherlands; 2 Corbin & Company, Maple Grove, Minnesota, USA

**Keywords:** Anterior cervical discectomy and fusion, Silicon nitride, Polyetheretherketone, PEEK, Interbody spacers, Randomized controlled trial, Herniated disc

## Abstract

**Background:**

Anterior cervical discectomy with interbody fusion cages is considered the standard surgical procedure in patients with cervical disc herniation. However, PEEK or metal cages have some undesirable imaging characteristics, leading to a search for alternative materials not creating artifacts on images; silicon nitride ceramic. Whether patients treated with silicon nitride ceramic cages have similar functional outcome as patients treated with PEEK cages is not known. We present the design of the CASCADE trial on effectiveness of ceramic cages versus PEEK cages in patients with cervical disc herniation and/or osteophytes.

**Methods/Design:**

Patients (age 18–75 years) with monoradicular symptoms in one or both arms lasting more than 8 weeks, due to disc herniation and/or osteophytes, are eligible for the trial. The study is designed as a randomized controlled equivalence trial in which patients are blinded to the type of cage for 1 year. The total follow-up period is 2 years. The primary outcome measure is improvement in the Neck and Disability Index (NDI). Secondary outcomes measures include improvement in arm pain and neck pain (VAS), SF-36 and patients' perceived recovery. The final elements of comparison are perioperative statistics including operating time, blood loss, length of hospital stay, and adverse events. Lateral plane films at each follow-up visit and CT scan (at 6 months) will be used to judge fusion and the incidence of subsidence. Based on a power of 90% and assuming 8% loss to follow-up, 100 patients will be randomized into the 2 groups. The first analysis will be conducted when all patients have 1 year of follow-up, and the groups will be followed for 1 additional year to judge stability of outcomes.

**Discussion:**

While the new ceramic cage has received the CE Mark based on standard compliance and animal studies, a randomized comparative study with the golden standard product will provide more conclusive information for clinicians. Implementation of any new device should only be done after completion of randomized controlled effectiveness trials.

## Background

Since the introduction of anterior approach to the cervical spine by Cloward [1] and Smith [2], a dispute has arisen about the best surgical treatment. The purpose of all anterior cervical surgical procedures is removal of the intervertebral disc in order to decompress the nerve root and alleviate radicular pain and/or myelopathy. However, cervical instability and segmental collapse with recurrent radicular pain has been documented after anterior discectomy. For this reason, most surgeons perform anterior discectomy with interbody fusion (ACDF) although there is controversy about the benefits of adding interbody fusion to the cervical discectomy technique [3-9].

Anterior discectomy with fusion (ACDF) using bone graft in dowel or block form can have complications associated with graft collapse and can be associated with pain at the donor site if the graft is harvested from the iliac crest [10]. Interbody fusion using metal or plastic cages to contain and reinforce bone graft has been shown to have several advantages over bone blocks [11]: 1) using a cage allows the surgeon to fill the space without harvesting bone from the iliac crest in most cases; local bone or a bone graft substitute can be used instead; 2) the strength of the cage material ensures preservation of the disc height; bone blocks can crumble, decreasing the disc height with consequent neuroforaminal stenosis; and 3) using a cage saves time in the operating room and reduces blood loss. If there is no iliac crest bone graft harvest, there is less postoperative pain, as well.

Both plastic and metal cages have disadvantages as well. The principal plastic material, polyetheretherketone (PEEK), is hydrophobic and a mild chronic fibrous tissue reaction develops around the implant [12]. PEEK is invisible on imaging and therefore dislodgement and subsidence are difficult to determine. The two most commonly used metals, stainless steel and titanium distort magnetic resonance imaging (MRI) and CT scans, making determination of fusion and evaluation of degeneration difficult[13,14]. At present, ACDF with a PEEK cage is considered the golden standard for cervical disc herniation by many surgeons [15,19].

**Figure 1:**
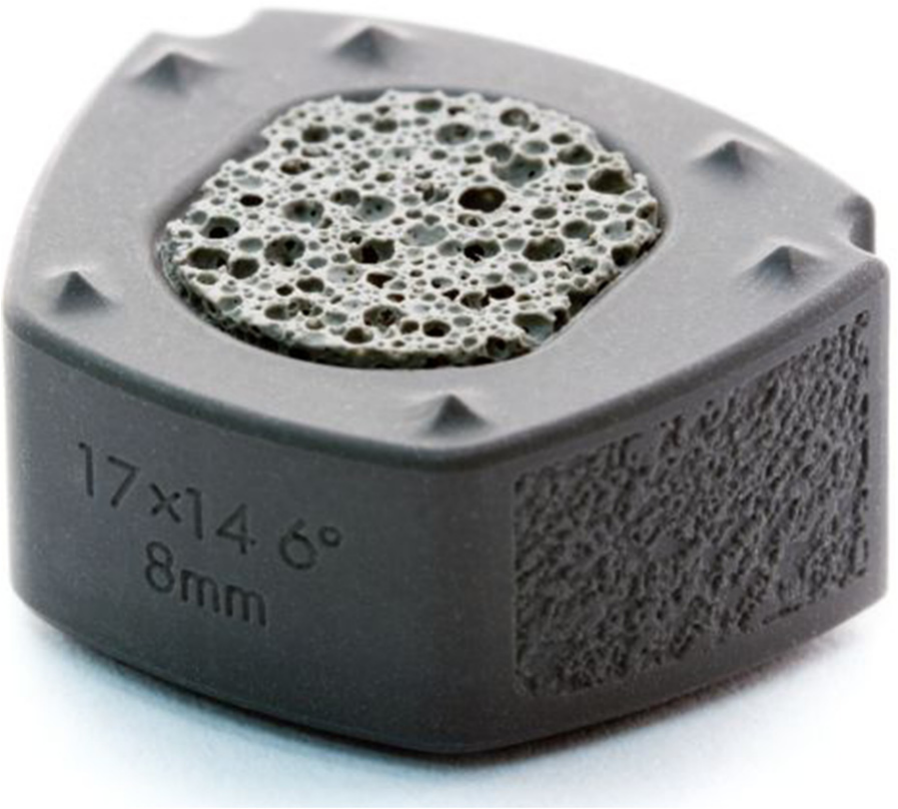
Valeo C+CSC cervical interbody fusion device.

**Figure 2:**
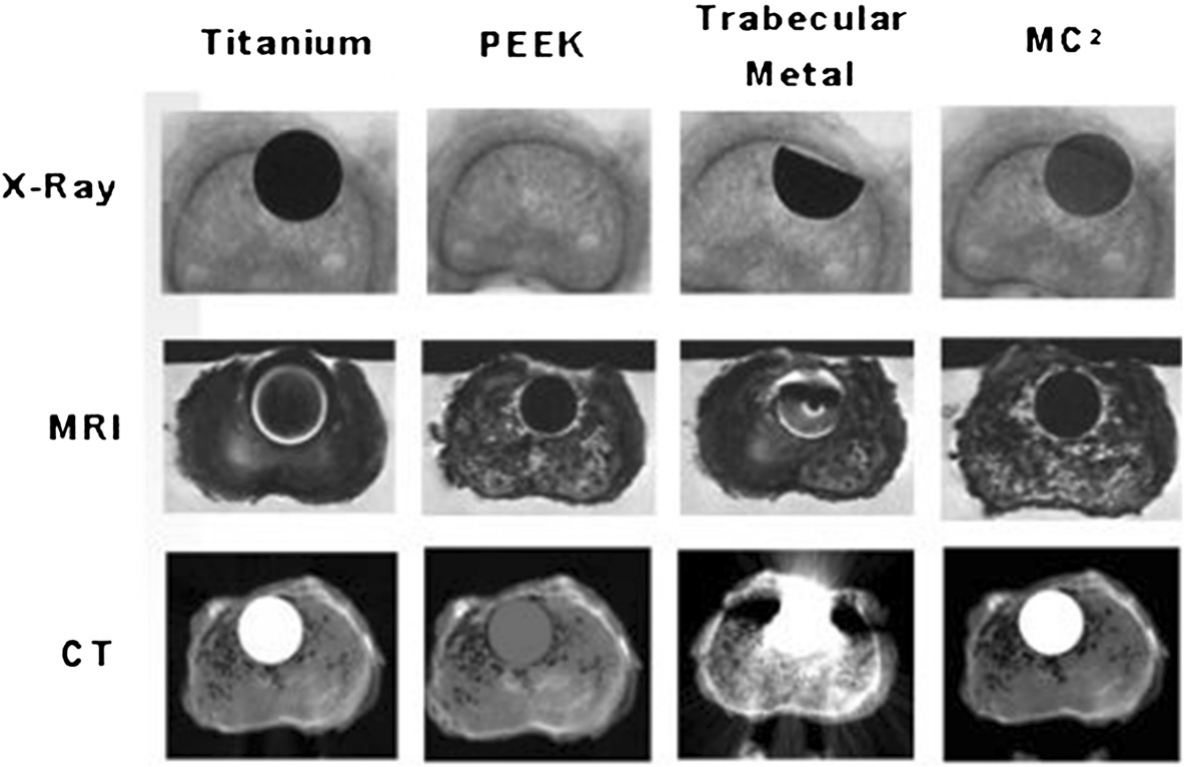
Silicon nitride, titanium, PEEK and trabecular metal imaging characteristics in a human cadaveric vertebra (unpublished data).

Recently, ceramic materials have been evaluated as alternatives for interbody fusion devices. The cortical ring of the new device is manufactured by study sponsor Amedica Corporation (Salt Lake City, Utah) from its proprietary “MC^2^” silicon nitride (Si_3_N_4_) ceramic. MC^2^ silicon nitride ceramic is a hydrophilic negative charged ceramic, which means that fluid (blood with nutrients) and proteins attach to the material, facilitating bone cell adherence and incorporation of the material in the surrounding bone. Cancellous Structured Ceramic (CSC) is a porous version of the same MC^2^ silicon nitride ceramic (Figure 1). Therefore, the entirety of this device is manufactured from identical material. The CSC material fills the center hole for the purpose of providing a scaffold for bone ingrowth. Because the surgical technique of this device does not involve harvesting autograft for packing into the pores of the porous trabecular structure, these implants afford the possibility of avoiding harvesting iliac crest autograft with attendant benefits of avoiding patient co-morbidities. Moreover, the silicon nitride material has desirable imaging properties. It is visible (like cortical bone) but does not create an artifact on CT or MR images (Figure 2).

The Valeo C+CSC cage has received the CE Mark based on preclinical testing and standards compliance, but has not been evaluated in a clinical study. Good clinical practice means that every new device should be compared to the golden standard prior to implementation of the device on a large scale. Therefore, it is necessary to perform a randomized controlled effectiveness trail on Valeo C+CSC versus PEEK cages. In the CASCADE (CAncelous Structured Ceramic Arthrodesis DEvice) trial, we will randomly and blindly compare anterior discectomy with ceramic cages versus anterior discectomy with PEEK cages. In this equivalence trial, we hypothesize similar effectiveness and no difference in clinical improvement between the ceramic cage and the PEEK cage as measured with the Neck Disability Index (NDI). Moreover, radiological properties will be documented focusing on fusion and subsidence.

## Methods/design

The study is designed as single center randomized controlled trial in which patients will be blinded for the allocated treatment. The follow-up period will be 2 years. All patients between 18 and 75 years old with monoradicular symptoms in one or both arms lasting more than 8 weeks are eligible for the trial. MRI must confirm cervical disc herniation and/or osteophytes in accordance with clinical symptoms. Additional inclusion and exclusion criteria are listed in Table 1.

**Table 1:** Selection criteria for trial eligibility

**Inclusion criteria**	
	• Age 18-75 years
	• Radicular signs and symptoms in one or both arms (i.e., pain, paresthesia or paresis in a specific nerve root distribution) or symptoms and signs of acute or chronic myelopathy
	• At least 8 weeks prior conservative treatment (i.e., physical therapy, pain medication)
	• Radiographic diagnosis of cervical disc herniation and/or osteophyte at 1 level (C3-C4 to C7-T1) in accordance with clinical signs and symptoms
	• Ability and willingness to comply with project requirements
	• Written informed consent given by the subject or the subject's legally authorised representative
**Exclusion criteria**		
	• Previous cervical surgery (either anterior or posterior)	
	• Increased motion on dynamic studies (> 3 mm)	
	• Severe segmental kyphosis of the involved disc level (> 7 degrees)	
	• Patient cannot be imaged with MRI	
	• Neck pain only (without radicular or medullary symptoms)	
	• Infection	
	• Metabolic and bone diseases (osteoporosis, severe osteopenia)	
	• Neoplasm or trauma of the cervical spine	
	• Spinal anomaly (Klippel Feil, Bechterew, OPLL)	
	• Severe mental or psychiatric disorder	
	• Inadequate Dutch language	
	• Planned (e)migration abroad in the year after inclusion	

Patients are referred by a neurologist with MRI of the cervical spine. During the first visit to the neurosurgical outpatient clinic, the patient’s history and a standard neurological examination will be documented. Based on our selection criteria, the neurosurgeon decides whether a patient is eligible for the trial.

### Informed consent and patients’ safety

The patient’s written informed consent is obtained. The patient will be notified that they are free to withdraw their consent at any time. An independent physician has been appointed to answer patient questions and monitor the study, when necessary. Adverse events or complications will be monitored and followed up until stable or resolved. The project will be conducted according to the guidelines of Good Clinical Practice. The study has been reviewed and approved by the Medical Ethics Committee (METC) of Southwest Holland.

### Randomization procedure

In order to eliminate confounding factors, the study is designed as a randomized controlled trial. Patients will be randomly allocated to the Valeo C+CSC or PEEK cages. Randomization will take place in the operating room within 6 weeks after inclusion. A randomization list was prepared by the data manager. A random numbergenerator was used to create the allocation sequence of blocks of 4, 6, and 8 to ensure equal distribution of the randomized treatments. The data manager, who is not involved in the selection and allocation of patients, prepared numbered, coded, sealed envelopes containing the treatment allocation. In the operating room, after induction of anaesthesia, the surgeon will open the next numbered envelope and the allocated treatment will be performed. Patients will be kept blinded for the allocated treatment for 1 year.

### Surgical intervention

All patients will be operated by the authors MA or JW, who both have extensive experience in cervical spine surgery and this ACDF technique. The patients will be positioned supine with their neck in neutral position or slightly extended under general anaesthesia. The affected cervical disc level will be verified with fluoroscopy. A small transverse incision will be made on the right side. Medial to the carotid sheath, the pre-vertebral space will be opened and the anterior cervical spine will be exposed. Caspar spreader and 2 distraction pins will be placed in the affected segment. A standard anterior discectomy with the aid of loupe magnification or microscope (depending on the surgeon’s preference) will be performed in all cases. The posterior longitudinal ligament will be opened and the nerve root and dura will be decompressed adequately. Once the anterior discectomy has been performed, a PEEK interbody cage (Medicrea Manta, Lyon, France) (group 1) filled with local bone[20] obtained from removal of osteophytes, or the Valeo C+CSC spacer (Amedica Corporation, Salt Lake City, Utah) (group 2) will be placed within the intervertebral space under fluoroscopic guidance. The CSC core should be smeared with blood obtained by scratching the end plate after the disc space is prepared. No supplemental fixation (e.g., cervical plate) will be used in the procedure. If required, a vacuum drain will be placed and the wound will be closed in layers.

### Outcome assessment

Baseline assessments include demographics, work status, smoking status, neck and arm pain, history of neck trauma, medical history, pain medication, body mass index, and neurological signs and symptoms. A general physical and neurological examination is performed before the patient is enrolled into the study and will be repeated at each subsequent visit.

We will assess the below described validated outcome parameters. Patients will not be informed about their previous scores. Follow-up examinations will take place by the surgeon at 3 months, 6 months, 12 months, and 24 months after randomization. The schedule of follow-up visits, radiographic studies and outcomes measures is included in Table 2. The following measures of outcomes will be used:

1) Neck Disability Index (primary outcome measure): The NDI is a patient-completed 10-item questionnaire on 3 different aspects; pain intensity, daily work related activities and non-work related activities. Each item is scored from 0 to 5 and the total score ranges from 0 (best score) to 50 (worst score). The NDI is a modification of the Oswestry Disability Index and has been shown to be reliable and valid for patients with cervical pathology [21,22] and has been validated in the Dutch language [23].

2) Short-Form 36 (SF-36): The SF-36 [24] is a generic health status questionnaire that can easily be filled out by the patient at home. The questionnaire consists of 36 items on physical and social status of the patient subdivided in 8 domains; 1) physical functioning, 2) physical restrictions, 3) emotional restrictions, 4) social functioning, 5) somatic pain, 6) general mental health, 7) vitality and 8) general health perception. The questions are scored on a scale of 0 (worst health) to 100 (ideal health). This questionnaire has been used frequently and is validated in surgical studies on spine pathology [25,26].The Dutch language version has also been validated [27].

3) Pain intensity, measured by Visual Analogue Score (VAS) of arm and neck. The VAS of arm pain will measure the experienced pain intensity in the arm during the week before visiting the researcher. Pain will be assessed on a horizontal 100 mm scale varying from 0 mm (no pain) to 100 mm (worst pain imaginable). Patients do not see the results of earlier assessments and will score the pain experienced at the visit. Since many patients with radicular arm pain have neck pain as well, we will also measure the intensity of solitary neck pain. Reliability, validity and responsiveness of VAS have been shown [28].

4) Perceived recovery: Likert Scale is a 7-point scale measuring the perceived recovery, varying from ‘complete recovery’ to ‘worse than ever’. This outcome scale has been used in previous studies and has been shown to be valid and responsive to change [29]. “Complete recovery” and “almost complete recovery” are defined as good result.

5) Radiological outcome: Anterior/posterior, and lateral views of the cervical spine will be performed at each visit. Displacement or subsidence of the device will be assessed by using the lateral radiograph. Only a change of >3 mm will be considered clinically significant due to the margin of error in radiographic determination of displacement distances. Radiolucency at the cage-bone interface will be assessed as either present (if extending 50% of the length of the cage) or absent using a standard lateral radiograph. To verify fusion, CT axial images at 2 mm slices will be taken at the 6 months visit only.

6) Other outcome measures: In addition to the outcomes measures, data will be captured on details of the surgical procedure and hospitalization such as type and size of implant, duration of surgery, estimated blood loss, operative and postoperative complications, day of mobilization, and duration of hospitalization. Adverse events and reoperations will also be recorded by the operating surgeon.

**Table 2:** Data collection and outcome measures

**Measure**	**Pre-Op**	**Intra-Op**	**3 months**	**6 months**	**12 months**	**24 months**
Inclusion / exclusion criteria	x					
Product use, operative time, blood loss		x				
Neurological examination	x		x	x	x	x
Neck Disability Index, Arm Pain VAS, Neck Pain VAS, SF-36, Recovery Likert	x		x	x	x	x
Radiographs X-ray	x		x	x	x	x
CT scan				x		

The de-identified data from the initial visits, hospitalization and follow-up visits are entered by research nurses and physician assistants into a database via an Electronic Data Capture system (Acumen Healthcare Solutions, LLC, Plymouth, Minnesota, USA). Hospital staff schedules follow-up visits and supervises the collection of the patient-completed data forms. The source documents are kept filed in the hospitals where the procedures are performed.

Participating patients are given a small stipend to cover travel costs for follow-up visits. The cost of the procedure (including the interbody fusion devices) is covered by the patient’s health insurance, but the expense of extra imaging and radiologist fees are paid by the study sponsor.

### Sample size and data analysis

Sample size for this equivalence trial has been established using power analysis incorporating data from journal article reports of similar ACDF studies. For sample size calculations, we have used the change in NDI from pre-op to one year post-op cited in the literature for ACDF with carbon fiber reinforced PEEK cages: 10% reduction (improvement) with a standard deviation of 22% [30]. The Minimal Clinically Important Difference (MCID) for the NDI is 7.5 points or 15% [31], which is the equivalence interval for sample size calculation. A 46-patient Valeo C enrollment has a power of .90 when compared with a PEEK study arm of the same size. Incorporating a one-year estimate of 8% loss to follow up, a total of 100 patients need to be enrolled.

The statistical significance of categorical data such as fusion will be tested using the Pearson Exact test. Parametric data will be evaluated using the Student’s t-test. The first data analysis will be performed when all of the 1-year follow-up data is available for the primary endpoint. The two groups will be considered equivalent if the mean NDI improvement for the silicon nitride cage group is within a range from the mean of the PEEK group minus the NDI MCID to the PEEK mean plus the MCID. A repeated measurements analysis of variance for the primary outcome measure will also be performed in order to compare the evolving patterns over time.

The inclusion period started in December 2011 and 2-year follow-up measures will be completed by the end of 2014.

## Discussion

A cervical radicular syndrome due to disc herniation is a well-known entity with an annual incidence rate of 83 per 100,000. Patients usually present with radicular arm pain and paraesthesiae, with or without neck pain. More than 90% of the patients have a favourable outcome with conservative treatment only [32] .Surgery is indicated whenever disabling pain persists. Anterior cervical discectomy (ACD) is the basic surgical treatment of patients with radicular pain caused by cervical disc herniation. In 1958, Cloward first described anterior cervical decompression with the use of autologous iliac crest interbody graft (ACDF) to maintain disc height [1]. Smith and Robinson developed a technique using iliac crest bone blocks that was the standard for many year [2].There is still controversy about the benefits of adding interbody fusion to the cervical discectomy technique [4,10,33,34]. Frequently surgeons perform ACDF to maintain disc height and cervical alignment, and promote bony fusion to prevent instability [25].

Various prospective randomised trials have been performed comparing anterior discectomy with additional interbody fusion [3-9]. These results suggest that interbody fusion may not be necessary in all cases. However, definite conclusions could not be drawn due to methodological flaws such as small sample size, non-homogenous patient population, undefined randomisation procedures and inconsistent outcome measures. Two large randomized trials are currently addressing the question of the value of adding fusion or cervical total disc replacements to the cervical discectomy procedure [35,36]. It is anticipated that ACDF will still be a viable choice for many patients even after these trials are completed.

Although there is no consensus on which interbody device to use, currently PEEK cages are considered as the golden standard for anterior cervical discectomy with fusion by many surgeons [15-19]. However, PEEK and titanium have some undesirable imaging characteristics that result in a demand for better cages with similar effectiveness. Figure 2 illustrates the superior imaging properties of MC^2^ ceramic material compared to PEEK, titanium and trabecular metal. Moreover, the CSC material fills the center hole for the purpose of providing a scaffold for bone ingrowth resulting in solid fusion and no subsidence of the cage. The CSC form of the material has performed well in an animal model [37] but the results and effectiveness need to be validated in humans. It is possible that solid fusion without signs of subsidence may lead to improved clinical outcome.

The present protocol of the CASCADE trial is designed to demonstrate the effectiveness and security of cancellous structured ceramic cages compared to the golden standard PEEK cages in patients treated with anterior cervical discectomy and fusion. Worldwide implementation of new devices should only be done after completion of randomized controlled trials. In our opinion, Level 1 evidence that government and health insurance organizations are demanding would not be possible without close cooperation between industry and researchers, but transparency is warranted and relevant conflicts of interest must be disclosed.

## Abbreviations

CASCADE: Cancellous structured ceramic arthrodesis device; ACDF: Anterior cervical discectomy with fusion; ACD: Anterior cervical discectomy; PEEK: Polyetheretherketone; CSC: Cancellous structured ceramic; MRI: Magnetic resonance imaging; CT: Computer tomography; NDI: Neck disability index; VAS: Visual analogue scale.

## Competing interests

Dr. Arts. Dr. Wolfs and Mr. Corbin are receiving financial support for their work on this study from Amedica Corporation.

## Authors’ contributions

MA designed the protocol, contributed to acquisition of data, is primary investigator and coordinator of the trial. JW has contributed to acquisition of data. TC has contributed to the case record forms, is responsible for the sample size calculation and analysis of the trial. All authors read and approved the final manuscript.

## Pre-publication history

The pre-publication history for this paper can be accessed here:

http://www.biomedcentral.com/1471-2474/14/244/prepub
